# Understanding Mumps Dynamics: Epidemiological Traits and Breakthrough Infections in the Population under 15 Years of Age in Jiangsu Province, China, 2023

**DOI:** 10.3390/vaccines12090986

**Published:** 2024-08-29

**Authors:** Mingma Li, Zhiguo Wang, Zhihao Liu, Xiuying Deng, Li Wang, Yuanyuan Zhu, Yan Xu, Lei Zhang, Yuanbao Liu, Bei Wang

**Affiliations:** 1 Department of Epidemiology and Health Statistics, School of Public Health, Southeast University, Nanjing 210009, China; 2 Department of Expanded Program on Immunization, Jiangsu Provincial Center for Disease Control and Prevention, Nanjing 210009, China; 3 Department of Health Education, Jiangsu Provincial Center for Disease Control and Prevention, Nanjing 210009, China

**Keywords:** mumps, epidemiology characteristics, breakthrough cases

## Abstract

Despite coverage of two doses of mumps-containing vaccines, mumps epidemics persist among children and young adults in China. This study aimed to analyze the epidemiological characteristics of mumps in Jiangsu Province, with a particular focus on breakthrough cases among high-incidence groups. Mumps cases reported in 2023 were systematically collected from the Infectious Disease Surveillance and Reporting System. A comprehensive descriptive epidemiological analysis was performed to elucidate the characteristics of the reported cases. A joinpoint regression (JPR) model was utilized to identify the temporal trends across various periods. Subsequently, immunization information for cases under 15 years of age was obtained through the Jiangsu Province Vaccination Integrated Service Management Information System to identify breakthrough cases and conduct exploratory analyses. A total of 4142 mumps cases were reported in Jiangsu Province in 2023, yielding an annual incidence rate of 4.86/100,000. A total of 81.75% of the cases were students and childcare children, and the gender ratio was 1.5:1 (male/female). The JPR model analysis of weekly reported cases identified five distinct trend segments (1st: 1–8, weekly percent change (WPC) = 26.67 *; 2nd: 9–28, WPC = 3.11 *; 3rd: 29–34, WPC = −5.31; 4th: 35–37, WPC = 15.48; 5th: 38–52, WPC = −4.06 *), and the gender subgroups demonstrated similar trends to the overall pattern. Notably, 89.14% (3692/4142) of the total cases were among individuals under 15 years, with 96.02% (3545/3692) having been vaccinated against mumps. The number of single-dose breakthrough cases (SdBCs) was approximately fourfold (2847/698) that of two-dose breakthrough cases (TdBCs). The main population composition of TdBCs was children aged 0–5 years old, and the classification was dominated by childcare children and scattered children. The median time interval between initial immunization and onset were shorter in TdBCs than in the SdBCs group, and the median time interval between the last immunization and onset was interestingly similarly shorter. However, these situations were interestingly reversed in 105 laboratory-confirmed breakthrough cases. Therefore, the current vaccination strategies have demonstrated tangible effectiveness in preventing and controlling mumps. However, the high incidence of breakthrough cases among high-risk pediatric populations indicates that mumps immunization strategies still deserve more attention and research for better herd protection.

## 1. Introduction

Mumps is a highly contagious infectious disease of the respiratory system caused by the mumps virus and characterized by parotid gland swelling, pain, and non-suppurative inflammation [1,2]. In severe cases, the mumps virus can invade the central nervous system through the bloodstream and lead to meningoencephalitis, and organs such as the pancreas, testes, ovaries, and thyroid are also susceptible to viral involvement; the incubation time averages 16–18 days with a range of 12–25 days [3,4,5]. Before the implementation of national routine mumps vaccination, nearly all children aged 5–9 years had serologic evidence of exposure to the virus [6]. In the past few years, outbreaks and recurring epidemics of mumps have surfaced in the United States and certain advanced European nations despite high vaccination coverage rates of Measles–Mumps–Rubella (MMR) vaccination [7,8,9,10,11]. In response to the need for enhanced protection against mumps, China monitored mumps as a Category C infectious disease through the National Notifiable Disease Reporting System (NNDRS) and implemented a policy in July 2020 to routinely administer two doses of the MMR vaccine to children aged 8 and 18 months, aiming to strengthen the protection against this infectious disease [12,13]. The proven efficacy of vaccines in mumps prevention underscores their critical role, yet ongoing optimization of targeted immunization strategies is needed to boost serum antibody levels and sustain robust antibody titers within the population [14,15,16]. In the wake of the recent global health challenges, continuously monitoring the evolving epidemiological landscape of mumps through meticulous analysis of disease surveillance data is paramount. Such efforts are crucial for the timely evaluation and adjustment of immunization strategies, as well as for pinpointing emerging risk factors that could precipitate future mumps outbreaks.

Therefore, in order to clarify the latest changes in the epidemiological features of mumps, this study systematically analyzed the mumps surveillance data from Jiangsu Province in 2023 to describe the epidemiological distribution in detail and conduct an exploratory analysis of the immunization history of the reported cases, with the ultimate goal of providing evidence for the evaluation of mumps vaccination strategies.

## 2. Materials and Methods

### 2.1. Data Collection and Management

**Figure 1:**
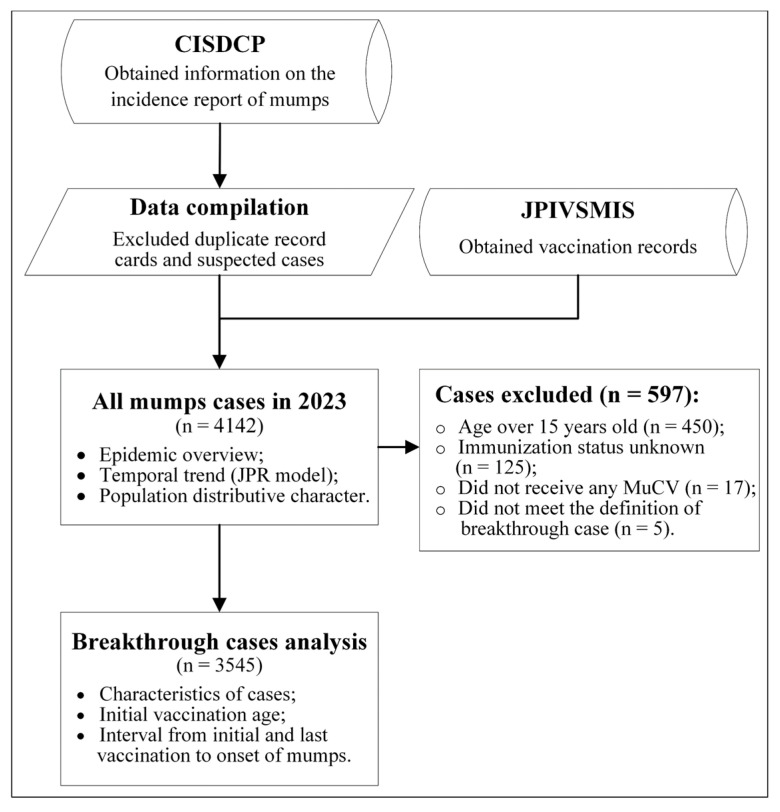
Flowchart of the research process. CISDCP: the China Information System for Disease Control and Prevention; JPIVSMIS: the Jiangsu Province’s Integrated Vaccination Service Management Information System; MuCV: Mumps-containing vaccines.

Data on mumps cases reported from January to December 2023 were obtained from the China Information System for Disease Control and Prevention (CISDCP). Physicians are mandated to report confirmed or suspected cases of mumps within a 24 h window through the online platform or via a standardized infectious disease notification form, with subsequent review conducted by qualified administrative personnel. The diagnosis of mumps cases adheres to the ‘Diagnostic Criteria for Mumps’, as stipulated by the Chinese Ministry of Health [17], encompassing a comprehensive evaluation of epidemiological exposure, clinical signs, and corroborative laboratory test outcomes. The dataset for each case report was meticulously aggregated, incorporating a spectrum of demographic specifics (residential identification numbers, gender, age, and occupation) alongside a comprehensive array of clinical particulars (initial symptomatic date, diagnostic confirmation date, respective diagnostic classifications). This study incorporated cases that were either clinically diagnosed or laboratory confirmed. Clinically diagnosed cases are determined by pain, tenderness, and swelling in one or both parotid glands, and (or) history of contact with mumps cases 14–28 days before onset. Laboratory-confirmed cases are those in which serological or molecular testing confirms mumps infection. Subsequently, we used the unique residential identification number to retrieve the vaccination records of the cases included from the Jiangsu Provincial Comprehensive Vaccination Service Management Information System (JPIVSMIS). Demographic data were culled from the Jiangsu Provincial Bureau of Statistics (https://tj.jiangsu.gov.cn/2023/index.htm, accessed on 12 June 2024). The flowchart in Figure 1 illustrates the general process of this study.

### 2.2. Descriptive Epidemiological Analysis for All Cases

The number of reported cases and incidence rate were calculated monthly and annually and stratified by gender and age groups. Graphical illustrations such as bar and line charts were used to represent trends in cases and incidence rates. We visualized the distribution of age and occupation in different case classifications through clustered bar charts. The occupational classification of the population is based on statistics from the Statutory Reporting of Infectious Diseases Case Report Card. Notably, childcare children are those who have not yet reached school age and are enrolled in group childcare institutions such as nurseries and kindergartens, generally between the ages of 3 and 6 years, while scattered children are those who are not enrolled in these institutions but are raised in the family, mostly under the age of 3 years. Generally speaking, children under 3 years of age in the community are the focus of health care management. Additionally, we created radar charts to reveal the seasonality of the incidence rates in different regions. To better understand the distribution of mumps cases and their seasonal characteristics in more regional detail, we divided Jiangsu Province into three distinct regions based on geographical location and sociodemographic characteristics: southern, middle, and northern region.

Data mining, cleaning, and management were conducted using Microsoft Office Excel 2021, while all analyses were performed in R 4.3.3 software with the exception of the joinpoint regressions.

### 2.3. Joinpoint Regression Model Analysis

The joinpoint regression (JPR) model was utilized to detect temporal trends in the weekly reported cases of mumps in Jiangsu Province for the year 2023. The fundamental concept involves using several change points to divide the entire study period into different intervals, followed by segmental regression and statistical modeling analysis for the incidence trends in each interval [18,19]. We conducted fitting analyses for the overall weekly case numbers as well as for subgroups stratified by gender. The standard parameterization of the JPR model for the observations $\left(x_{1},y_{1}\right),\cdots ,(x_{n},y_{n})$, where $x_{1}\leq \cdots \leq x_{n}$ without loss of generality, can be written as

$$E\left(y|x\right)=\beta_{0}+\beta_{1}x+\delta_{1}{\left(x-\tau_{1}\right)}^{+}+\cdots +\delta_{k}{\left(x-\tau_{k}\right)}^{+}$$

where $E\left(y|x\right)$ is continuous at $\tau_{k}$ under the constraint of $\alpha^{+}=\alpha$ for α>0 and 0 otherwise. y represents the variable of interest, x denotes the calendar week, $\beta_{1}$ is the coefficient in the regression model, $\delta_{k}$ corresponds to the coefficients of the piecewise function within section k, and $\tau_{k}$ are the unidentified junction points.

We employed the grid search method (GSM) to analyze the number, position of turning points, and model parameters. Utilizing the modified Bayesian information criterion (MBIC) to select the most appropriate model [18,20], we subsequently calculated the weekly percent change (WPC) and 95% confidence interval (CI) for the number of cases in each trend segment. The *Z* test was used to assess whether WPC was significant (*p* < 0.05), and the tendencies were characterized as rising or falling when the WPC was on the positive or negative side, respectively. Conversely, the trends were deemed to be stable when the WPCs did not exhibit significant values (*p* ≥ 0.05). Joinpoint regression analysis was executed using the Joinpoint Regression Program, Version 5.1.0.0.

### 2.4. Analysis of Immune Status in Breakthrough Cases

This study’s exploratory analysis of breakthrough cases focuses on pediatric cases aged under 15 years. The breakthrough case is defined as the case in which a patient received at least one dose of MuCV 42 days before the onset of mumps symptoms (1.5-fold extended mumps virus incubation period) [21]. Cases in which the onset of illness occurred more than 30 days after the second dose of MuCV were classified as TdBC, whereas cases in which the onset of illness did not occur more than 30 days into the observation period after the second dose of MuCV were still classified as SdBC. We conducted a series of descriptive analyses on the distribution of breakthrough cases, calculating the vaccination age and time interval between immunization and mumps onset in months. We also calculated the interval between the initial and last immunizations for TdBCs.

## 3. Results

### 3.1. Overview of Cases

**Figure 2:**
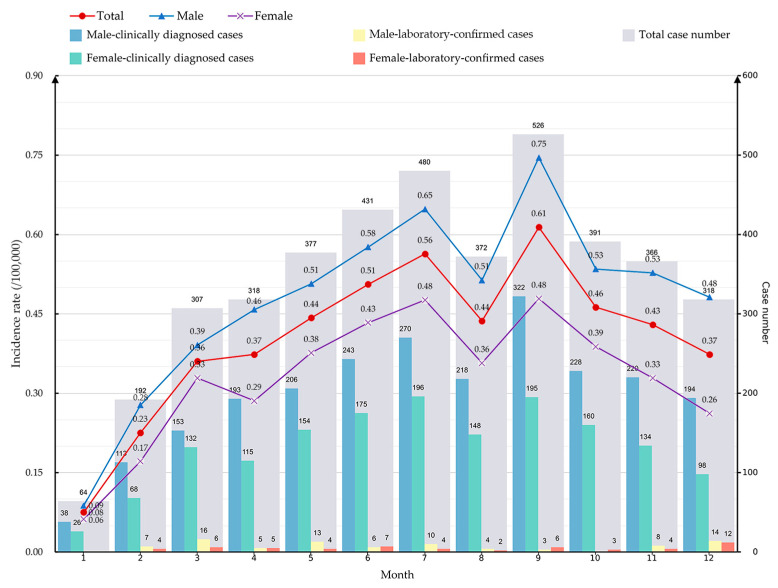
The incidence cases and rates of reported mumps in Jiangsu Province during 2023. The three-colored line charts depict the monthly incidence trends for the total population, males, and females (per 100,000). The composite clustered bar chart illustrates the monthly reported number of cases, differentiated by gender and case type.

A total of 4142 mumps cases were reported in Jiangsu Province during 2023 with an annual incidence of 4.86 per 100,000. And 3999 (96.50%) were clinically diagnosed cases, while 143 (3.50%) were laboratory-confirmed cases. The number of reported cases in the southern, mid, and northern region accounted for 48.24%, 20.14%, and 31.63% respectively, and the incidence rate of regional subgroups was 5.18, 4.95, and 4.39 per 100,000, respectively. Figure 2 presents the overall and gender-stratified monthly incidence rate of mumps cases, along with the number of reported cases and a breakdown by gender and case type.

### 3.2. Population Distributions of Mumps Cases

**Figure 3:**
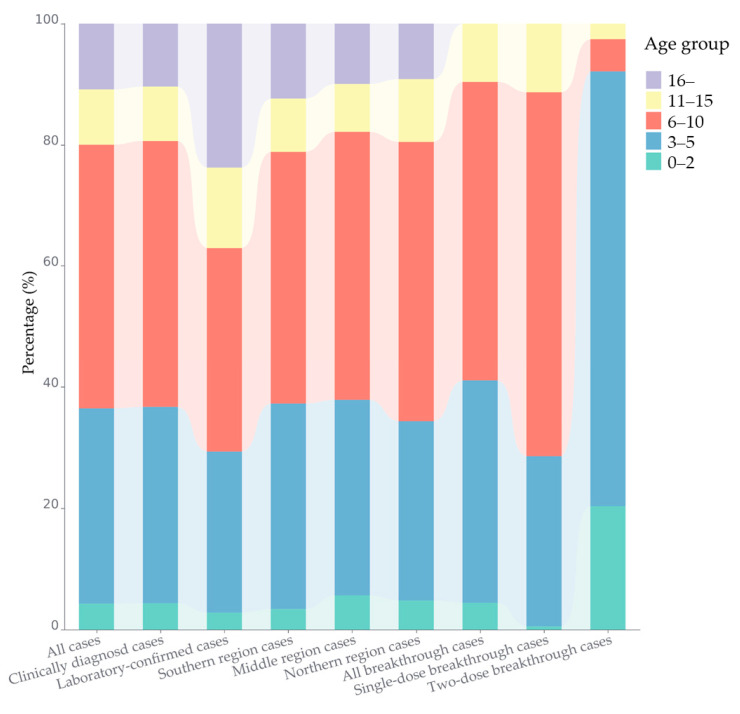
The proportion distribution of age groups of mumps cases by category in Jiangsu Province in 2023.

Throughout 2023, the reported number of cases and incidence rates of mumps were consistently higher in males compared to females (Figure 2). Specifically, the annual reported incidence rates were 5.75 and 3.95 per 100,000 for males and females, respectively, resulting in a male-to-female ratio of 1.50. Cases aged 3–5 and 6–10 years accounted for 32.18% (1333/4142) and 43.53% (1803/4142) of all cases, respectively, and were the main age distribution groups. This distribution pattern is similar across both clinically diagnosed and laboratory-confirmed cases, as well as in the three regions of Jiangsu (Figure 3). Moreover, there are a total of 143 laboratory-confirmed cases, of which 76.22% (109/143) are under the age of 15.

**Figure 4:**
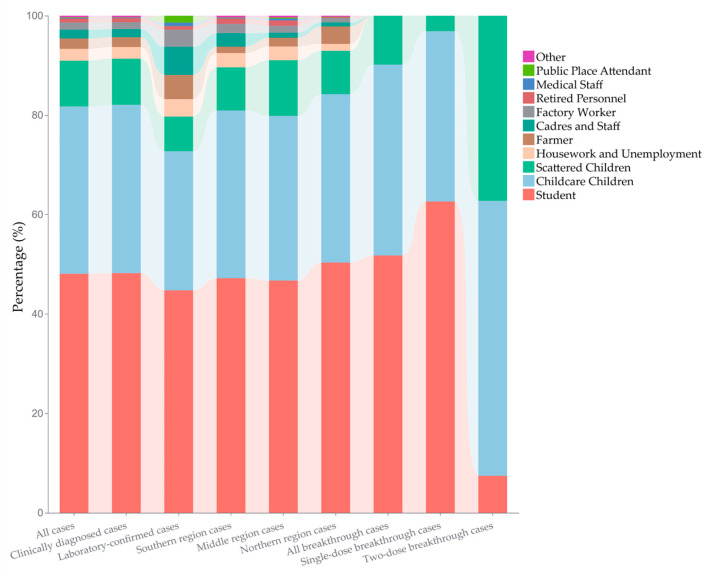
The proportion distribution of population classification of mumps cases by category in Jiangsu Province in 2023.

The vast majority of these cases were students, childcare children, and scattered children, accounting for 48.09%, 33.66%, and 9.22%, respectively. A slight decline in the proportion of the three occupational classifications was observed among the laboratory-confirmed cases, standing at 44.76%, 27.97%, and 6.99%, respectively. In addition, the proportion of student cases in the northern region (50.34%, 657/1305) was notably higher compared to the middle (46.72%, 392/839) and southern (47.20%, 943/1998) region of Jiangsu; see Figure 4.

### 3.3. Temporal Trends and Seasonal Patterns

**Figure 5:**
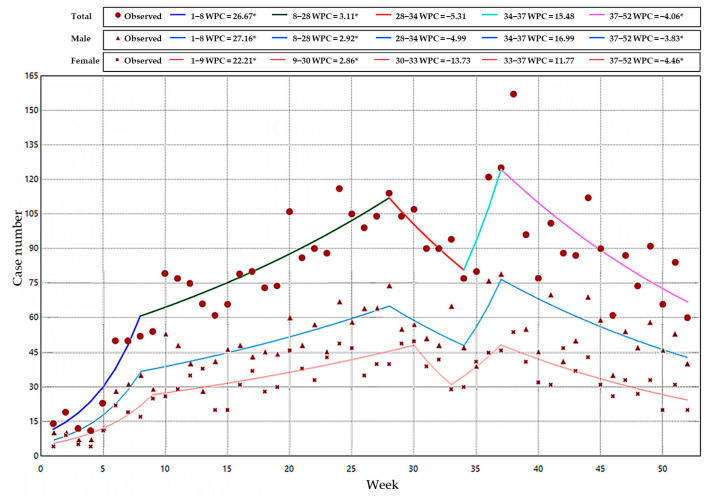
Weekly reported case number analysis using the joinpoint regression model for overall and gender-specific subgroups. WPC: weekly percent change.

The monthly incidence rate showed a bimodal trend of first increasing and then falling, and peaked in July (0.56 per 100,000) and September (0.61 per 100,000), respectively (Figure 2). This temporal pattern is further substantiated by the detailed joinpoint regression analysis conducted on the weekly reported case counts, as depicted in Figure 5. Four connection points were identified to divide the trend into five distinct segments (1st: 1–8, WPC = 26.67 *; 2nd: 9–28, WPC = 3.11 *; 3rd: 29–34, WPC = −5.31; 4th: 35–37, WPC = 15.48; 5th: 38–52, WPC = −4.06 *; * indicates that the WPC is significantly different from zero at the α = 0.05 level). The patterns derived from the JPR analysis for the gender-specific subgroups are similar to the overall trend, except that females reach the lowest turning point earlier than males for one week in August and begin a second upward trend.

**Figure 6:**
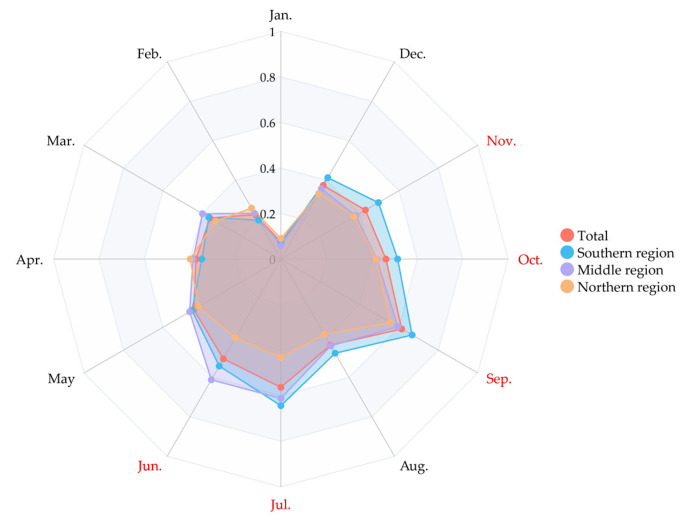
The seasonal distributions of incidence rates for mumps in Jiangsu Province and its three regions during 2023. Notes: Red represents months with a relatively high incidence rate.

The seasonal patterns of mumps exhibited slight variations across different regions of Jiangsu (Figure 6). From the radar charts, the overall and regional incidence rates show an obvious bimodal seasonal pattern, but the peaks in summer are lower in the northern region than those in other regions. Specifically, the incidence rate remains at 0.4 per 100,000 from April to July in the northern region. But the incidence rate in the other regions is significantly higher, especially in June and July, with the highest rate in the southern region in July (0.64 per 100,000).

### 3.4. Characteristics of Breakthrough Cases

**Figure 7:**
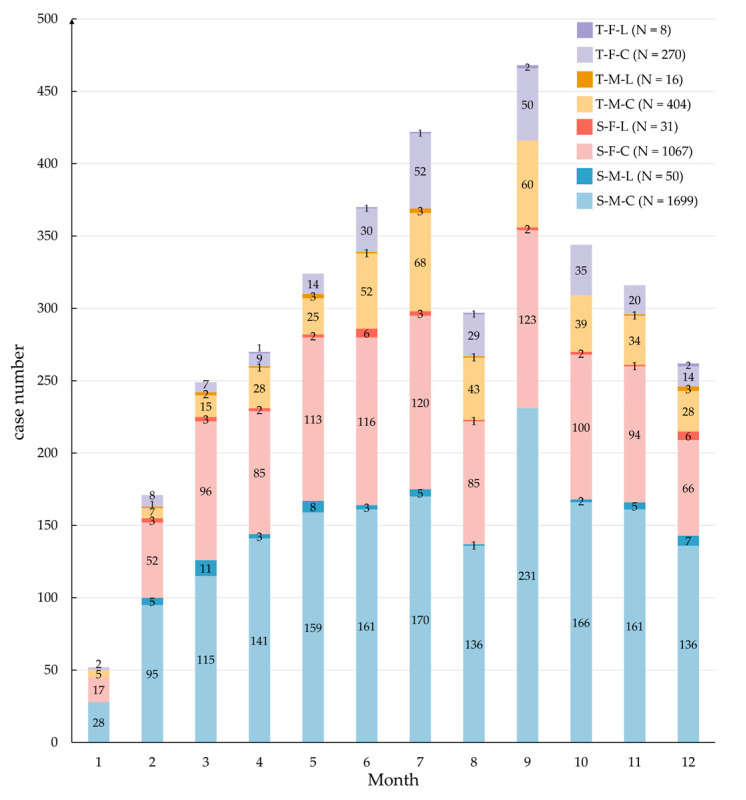
Distribution of breakthrough cases of mumps in Jiangsu Province during 2023. T: two-dose breakthrough case; S: single-dose breakthrough case; F: female; M: male; L: laboratory-confirmed cases; C: clinically diagnosed cases; N: number of cases throughout the year.

A total of 3692 cases were reported among individuals aged 0–15 years, with 96.02% (3545/3692) breakthrough cases over the whole province in 2023. Among these breakthrough cases, 97.04% (3440/3545) are clinically diagnosed cases, and 80.31% (2847/3545) are categorized as single-dose breakthrough cases (SdBCs). The male-to-female ratio is 1.60 (2169/1376), and more detailed distribution information of breakthrough cases is presented in Figure 7. Geographically, the distribution of breakthrough cases in the southern, middle, and northern regions of Jiangsu is 47.53%, 20.56%, and 31.90%, respectively. Notably, in southern Jiangsu, two-dose breakthrough cases (TdBCs) constituted a significant proportion at 52.72% (368/698) of all TdBCs. The population classification and age distributions of SdBCs and TdBCs differed considerably, with the former predominantly being students (62.63%, 1783/2847), childcare children (34.25%, 975/2847) and aged 3–10 years (88.13%, 2509/2847), while the TdBCs were mainly childcare children (55.30%, 386/698), scattered children (37.25%, 260/698) and aged 0–5 years (92.12%, 643/698) (referenced in Figure 3 and Figure 4).

Table 1 presents a comparative analysis of immunization history and symptom onset among breakthrough cases. It demonstrates that the TdBCs group had a more concentrated distribution of ages at immunization and onset, as well as shorter intervals between immunization and onset, compared to the SdBCs group. Specifically, the median time elapsed between initial vaccination and onset is shorter for TdBCs (36.52, IQR (interquartile range): 17.87, in months) than for SdBCs (68.23, IQR: 42.14, in months), indicating a potentially different temporal profile between the two groups. Furthermore, within the TdBCs group, the median interval from the last vaccination to mumps onset was 26.43 months, with an IQR of 17.95 months.

There were 105 laboratory-confirmed breakthrough cases (L-BCs) under the age of 15, while the numbers of SdBCs, TdBCs, male and female were 81, 24, 66, and 39, respectively (Figure 7). The median ages at onset (in months) of all L-BCs, L-SdBCs, and L-TdBCs were 80.80 (IQR = 51.19), 80.67 (IQR = 54.12), and 86.65 (IQR = 46.97), respectively. And their median time intervals (in months) from initial immunization to onset were 62.07 (IQR = 47.12), 60.33 (IQR = 47.58), and 68.21 (IQR = 42.36), respectively. Moreover, the median time interval from the last immunization to the onset of L-TdBCs was 68.21 (IQR = 47.01 in months). More data details can be found in Table 1.

## 4. Discussion

The global COVID-19 pandemic and its associated public health and social measures (PHSMs) continue to influence the epidemiological characteristics of other infectious diseases and remind us of the need for constant vigilance. The heightened public willingness to participate in preventive health measures during the COVID-19 pandemic has emerged as a salutary consequence of the enhancement of disease prevention and control strategies [22]. Through diverse educational initiatives, individuals have garnered a profound comprehension of infectious disease mitigation and acknowledged the critical role of vaccination [23]. This enhanced awareness has, in turn, bolstered the coverage and uptake of the mumps-containing vaccine (MuCV). Mumps virus is known for its droplet transmission, leading to a broad geographic dispersion and recurrent outbreaks across regions [1,6,24]. This underscores the importance of comprehensive public health strategies that take into account the ubiquitous threat posed by this easily transmissible pathogen and that keep abreast of the latest epidemiological developments and influencing factors of the disease.

The principal objective of this study was to elucidate the demographic, temporal, and seasonal patterns characteristics of mumps using recent surveillance data. Additionally, we conducted a more comprehensive analysis of diagnosis categories, further gender distribution, and immunization histories that we did not cover in our previous study. The results indicate that the annual incidence of mumps in Jiangsu decreased significantly to 4.86 per 100,000 compared with the 2020 incidence rate of 8.58 per 100,000 population [25]. This progressive decline can be attributed to multifaceted efforts, with comprehensive preventive and control strategies for mumps in terms of high coverage of double-dose immunization, improved morbidity surveillance and rapid emergency response management [26,27].

A noteworthy alteration in the epidemiological profile of mumps within Jiangsu Province, contrasting with our preceding investigation, is the redistribution of high-incidence zones. Our previous study found that from 2017 to 2020, the hotspots with higher annual incidences of mumps are concentrated in the northern Jiangsu [25]. Conversely, the results of this study disclosed that the annual incidence rate in the southern region (5.18 per 100,000) markedly outstrips that in the mid (4.95 per 100,000) and northern regions of Jiangsu (4.39 per 100,000). The seasonal distribution illustrated in Figure 6 also underscores the strikingly lower monthly incidence rate in the northern region when compared with its mid and southern counterparts. This geographical shift in high-incidence areas can potentially be ascribed to the province-wide enhancement of two-dose MuCV coverage and immunization rates. Consequently, the principal driver of mumps outbreaks has transitioned from inadequate seroprevalence to increased susceptibility in densely populated regions. The observed disparities in the age and occupational profiles of affected individuals across the three regions suggest that the etiological factors leading to the respective outbreaks may not be identical. This necessitates further in-depth exploration to identify and elucidate the multifaced underlying determinants.

Through joinpoint regression analysis and monthly seasonal distribution, this study more comprehensively identifies the temporal epidemiological trends and seasonal patterns of mumps within a natural year. It is remarkable to observe that the secondary peak of the mumps epidemic season in Jiangsu during 2023 occurred from September to November, a period slightly antecedent to the national peak typically witnessed from November to January [28]. This temporal discrepancy might plausibly be attributed to underlying sociodemographic characteristics and variances in meteorological conditions across distinct regions. For example, in the former case, a large number of students return to school after the summer holidays, and the rise in population congregation density increases the risk of exposure to the virus and thus may create the second peak in September. Prior studies have elucidated that ambient temperature and relative humidity can exert an influence on mumps incidence rates by modulating population behavior and affecting the viability of the virus in environmental conditions [29,30,31]. The divergent seasonal distributions observed in the three regional subgroups of this study lend credence to the potential role of these meteorological factors in shaping the epidemiological profile of mumps. Furthermore, the outcomes of this study revealed that, whilst temporal trends in morbidity mirrored each other across genders, male morbidity consistently surpassed female morbidity at various junctures. The underlying biological mechanisms accounting for this sex-specific incidence pattern remain enigmatic. This may be attributable, in part, to the propensity for males to engage more frequently in outdoor group activities—a factor that could potentially augment exposure risks as per their behavioral patterns.

The progressive advancement of diagnostic capabilities and surveillance systems, particularly the enhancement of diagnostic proficiency, has bolstered our monitoring and management of mumps [26,32]. Nevertheless, mumps is Category C notifiable disease in China, with a markedly lower proportion of laboratory-confirmed cases amongst all reported instances compared to other respiratory infections—namely, seasonal influenza, measles, rubella, and pertussis [28]. Of the 4142 documented cases in this investigation, merely 3.5% (n = 143) were laboratory-confirmed. Clinical diagnoses are predicated solely on symptomatology and the history of suspected exposure, which might prompt less experienced clinicians to erroneously report illnesses presenting with akin symptoms or bacterial parotitis directly to the NNDRS. This could lead to an overestimation of mumps prevalence and potentially squander healthcare resources unnecessarily [33]. Accordingly, there is a compelling case for augmenting the laboratory confirmation of reported cases to paint a more accurate epidemiological portrait of mumps.

Most interestingly, our retrospective analysis revealed that 85.59% (3545/4142) of the reported cases had a history of vaccination with the MuCV. Our findings indicated that the number of SdBCs was approximately quadruple that of TdBCs (2847 vs. 698), mirroring patterns observed in previous assessments of MuCV efficacy [15,16,34]. Given the relatively limited protective effect of a single dose of MuCV, antibody titers exhibit a pronounced decline with advancing age, thus rendering breakthrough infections commonplace. Nonetheless, the presence of 19.69% TdBCs under the age of 15 underscores the insufficiency of the current two-dose immunization strategy to fully curb mumps. Despite the administration of two doses of MuCV, morbidity persists due to immune failure or waning antibody levels beneath protective thresholds. Ongoing evaluation of MuCV immunization, focusing on antibody persistence and cross-strain protective capacity, emerges as a pivotal concern in mumps prevention [35,36]. The observed disparities in age and demographic composition between TdBCs and SdBCs suggest that considering the recommendation for two-dose MMR vaccination in the National Immunisation Programme internationally, the epidemiological profile of mumps may progressively skew towards younger demographics. This trend warrants consideration by healthcare authorities when devising and executing disease preventive measures, encouraging the adoption of targeted interventions to address this evolving pattern.

We analyzed 105 laboratory-confirmed breakthrough cases and found that the age of onset was older in cases that had received two doses of MuCV, and the time interval from initial and last immunization to the onset of mumps was longer than in cases that had received only a single dose of MuCV. These results may suggest, to some extent, that two doses of MuCV provide longer immune protection than a single dose, as many previous studies have demonstrated experimentally and in immunological observational cohorts [37].

Several limitations need to be discussed in this study. Firstly, the diagnosis of mumps in surveillance relied on clinical diagnosis in the absence of laboratory confirmation. This may lead to misreporting of some cases of non-mumps virus infections with similar symptoms, thereby overestimating the prevalence of mumps, especially when performing breakthrough case identification. Secondly, we collected information on the immunization history of mumps cases but lacked vaccination for the general population; it was not possible to accurately estimate the protective efficacy of the MuCV. Despite the potential limitations described above, we posit that these limitations did not significantly influence the main findings of our study. The description of the immunological history of the reported cases in this study is valuable information that has been difficult to obtain in previous epidemiological analysis studies. The epidemiological dynamics obtained through timely and comprehensive analyses are instructive for the prevention and control of mumps.

## 5. Conclusions

Since the incorporation of a two-dose MMR vaccination schedule into the National Immunisation Programme, the control of mumps in Jiangsu Province has witnessed striking advancements. 

Several noteworthy alterations in epidemiological dynamics are evident, encompassing a reduction in reported incidence rates, geographic displacement of high-prevalence zones toward the southern regions of Jiangsu, an advancement in the timing of the secondary epidemic peak, and a demographic shift toward younger age groups experiencing heightened incidence. Moreover, the prevalence of breakthrough infections underscores the necessity for further exploration and refinement of vaccines and immunization strategies capable of conferring more enduring protection.

## Figures and Tables

**Table 1:** Distribution of onset and duration of immunization for different classifications of breakthrough cases.

Breakthrough Groups	Minimum	Q 1	Median	Q 3	Maximum	Mean	IQR
SdBCs (N = 2847)							
Age at onset, in months	11.30	70.31	87.17	112.13	194.63	93.05	41.82
Age at initial vaccination, in months	3.57	18.33	18.50	19.03	172.23	19.22	0.70
Months between initial vaccination to onset	2.60	51.09	68.23	93.23	176.23	73.82	42.14
TdBCs (N = 698)							
Age at onset, in months	19.63	38.24	45.35	56.60	193.43	51.29	18.36
Age at initial vaccination, in months	2.00	8.20	8.43	9.36	67.97	10.11	1.16
Age at last vaccination, in months	12.20	18.37	18.63	19.57	129.77	22.39	1.20
Vaccination interval, in months	1.33	9.80	10.20	10.73	111.33	12.28	0.93
Months between initial vaccination to onset	10.10	29.58	36.52	47.45	173.27	41.18	17.87
Months between last vaccination to onset	1.27	18.80	26.43	36.75	139.53	28.90	17.95
L-BCs (N = 105)							
Age at onset, in months	21.77	57.29	80.80	108.47	179.17	86.87	51.19
Age at initial vaccination, in months	8.10	18.23	18.40	18.94	34.13	17.16	0.70
Months between initial vaccination to onset	12.17	42.44	62.07	89.55	160.67	69.71	47.12
L-SdBCs (N = 81)							
Age at onset, in months	21.77	54.35	80.67	108.47	179.17	86.02	54.12
Age at initial vaccination, in months	8.10	16.98	18.43	19.02	34.13	16.98	2.04
Months between initial vaccination to onset	12.17	41.97	60.33	89.55	160.67	69.04	47.58
L-TdBCs (N = 24)							
Age at onset, in months	23.73	62.52	86.65	109.49	158.57	89.74	46.97
Age at initial vaccination, in months	8.17	18.27	18.32	18.62	20.50	17.76	0.34
Months between initial vaccination to onset	15.30	48.83	68.21	91.19	138.07	71.99	42.36
Months between last vaccination to onset	4.27	44.18	68.21	91.19	138.07	71.09	47.01

Note: SdBCs: single-dose breakthrough cases; TdBCs: two-dose breakthrough cases; L-BCs: laboratory-confirmed breakthrough cases; L-SdBCs: laboratory-confirmed single-dose breakthrough cases; L-TdBCs: laboratory-confirmed two-dose breakthrough cases; IQR (interquartile range) = Q3–Q1.

## Data Availability

The data that support the findings of this study are available from the CISDCP, but restrictions apply to the availability of these data, which were used under license for the current study. Data are available from the authors under reasonable request and with permission of the Jiangsu Provincial Center for Disease Control and Prevention.
